# Depression symptoms 6 years after stroke are associated with higher perceived impact of stroke, limitations in ADL and restricted participation

**DOI:** 10.1038/s41598-022-11097-9

**Published:** 2022-05-12

**Authors:** Charlotte Ytterberg, Linda Cegrell, Lena von Koch, Maria Wiklander

**Affiliations:** 1 Department of Neurobiology, Care Sciences and Society, Karolinska Institutet 23100, 141 83 Huddinge, Sweden; 2 Karolinska University Hospital, Stockholm, Sweden; 3 Department of Physiotherapy, Capio S:t Görans Hospital, Stockholm, Sweden

**Keywords:** Stroke, Depression, Rehabilitation, Outcomes research

## Abstract

Late post-stroke depression symptoms are understudied. This study aimed to investigate depression symptoms 6 years after stroke, and associations with perceived impact of stroke, activities of daily living (ADL), and participation in social and everyday activities. Data was collected in a 6-year follow-up in a longitudinal study of stroke. Assessments included Hospital Anxiety and Depression Scale (HADS) for depression symptoms, Stroke Impact Scale 3.0. for perceived impact of stroke, Barthel Index for ADL, Frenchay Activities Index for participation in social and everyday activities. The research questions were addressed by bivariate analyses (with HADS-D ≥ 4 as cut-off), and hierarchical multiple regression analyses using continuous HADS-D scores. Forty percent of the 105 participants (57% men, age 30–91) showed depression symptoms (HADS-D ≥ 4). Depression symptoms were associated with higher perceived impact of stroke, more dependence in ADL, and more restrictions in participation in social and everyday activities. Most of those with depression symptoms had low scores on HADS, indicating that even mild depression symptoms might be relevant to identify and target in treatment and rehabilitation of long-term consequences of stroke.

## Introduction

Stroke is a major cause of death and disability worldwide, affecting more than 13 million persons every year^1^. Improved acute treatment of stroke in the last decades, with increasing survival rates, calls for efficient strategies for post-stroke rehabilitation. The rehabilitation often occurs in the year following the stroke, with no or few guidelines for longer follow-up. Yet, long-term consequences of stroke are common, which might impact activities of daily living (ADL), and participation in the society^2–4^. Therefore, long-term consequences of stroke and their interrelationships are relevant to identify, to find targets for treatment and rehabilitation. This study adheres to the International Classification of Functioning, Disability and Health (ICF)^5^, which is based on the biopsychosocial model of disability. In the ICF activity is defined as “the execution of a task or action by an individual” and participation as “involvement in a life situation”.

Depression is more common after stroke^6^ than in the general population^7^ and may contribute to poorer quality of life and functioning^8–10^. Previous research has reported both early and late onset of post-stroke depression (PSD), with the highest prevalence during the first year^6,8^. The aetiology of PSD is still poorly understood but considered to be multifactorial, with biological and psychosocial components contributing to the development of depression symptoms^10^.

In recent years, an increased attention has been given to evaluating the efficacy of treatments of PSD^10^. Furthermore, different interventions to prevent PSD have been tested, including pharmacological, psychological, and non-invasive brain stimulation treatments, but the current evidence for efficacy of any of these treatments is weak^11^.

The prevalence of PSD has been reported up to 15 years^12^, but there is still a scarcity of research including factors associated with PSD in a longer time perspective. As post-stroke depression has been related to worse functioning^10^, relevant areas to investigate include perceived impact of stroke, activities of daily living (ADL) and participation in social and everyday activities.

Therefore, the aims of this study were to investigate depression symptoms 6 years after stroke, and associations with perceived impact of stroke, ADL, and participation in social and everyday activities.

## Methods

### Participants

Participants from the longitudinal study “Life After Stroke Phase 1” (LAS-1), who took part in a 6-year follow-up, were eligible for inclusion. The LAS-1 was a prospective observational study on the rehabilitation process after stroke, described in detail elsewhere^13^. From an original sample of 349 patients diagnosed with stroke, consecutively recruited years 2006–2007 at stroke units at Karolinska University Hospital in Stockholm, Sweden, 183 persons who were still alive were approached by mail for participation. Informed signed consent was obtained from all participants. Ethical permission for the original study and the 6-year follow-up was granted by the Regional Ethics Committee in Stockholm (2011/1573-32, 2012/428-32), and procedures were conducted in accordance with the Declaration of Helsinki.

In the current study, all participants from the LAS-1 study who consented to participate in the 6-year follow-up and who had completed the Hospital Anxiety and Depression Scale (HADS)^14^ were included.

### Procedure

Data was collected with structured face-to-face interviews by experienced occupational therapists and physiotherapists. The interviews were in most cases conducted in the participant’s home. If needed, a next-of-kin was present during the interviews.

### Data collection

Sociodemographic data was collected at baseline, within 5 days after stroke, and at the 6-year follow-up. The Barthel Index (BI) has shown good agreement with other stroke severity measures^15^ and was used to classify stroke severity at baseline. A score < 15 was classified as severe, 15–49 moderate, and ≥ 50 as mild stroke^16^. Cognitive function was assessed with the Mini-Mental State Examination (MMSE)^17^. One item from the Scandinavian Stroke Scale was used to assess presence and severity of aphasia at onset^18^. At 6 years the following data were collected.

#### Hospital Anxiety and Depression Scale

Depression symptoms were collected by the Hospital Anxiety and Depression Scale (HADS), which is a 14-item self-rating scale to screen for anxiety and depression among persons with physical ill-health^14^. The items cover non-physical symptoms of anxiety and depression, with seven items covering anxiety (HADS-A) and seven items covering depression (HADS-D). The respondent is asked to rate agreement with the statements for a period of 1 week, on a four-point scale ranging from 0 = no symptom to 3 = maximum symptom. The maximum score on HADS-D is 21 and a commonly used cut-off for depression is > 8^19^. However, it has been suggested that a lower score is more accurate to detect depression among persons with stroke^20^ and a cut-off of 4 was therefore used in this study.

#### Stroke Impact Scale

Perceived impact of stroke was assessed with the Stroke Impact Scale (SIS) version 3.0^21^, a 59-item scale assessing eight domains: strength, hand function, ADL, mobility, communication, emotion, memory and thinking, and participation. Responses on each domain are transformed into a 0–100 score, where 0 indicates maximal and 100 no perceived impact of the stroke. The scale also contains a single item reflecting perceived recovery from the stroke, which is rated on a visual analogue scale with a range of 0–100, where 0 reflects no, and 100 maximal, recovery.

#### Barthel Index

The Barthel Index^15^ was used to assess ADL. The instrument consists of ten questions about activities regarding personal care and mobility. The total sum score is 0–100 where higher scores indicate more independence. Any score below 100 indicates some level of dependence.

#### Frenchay Activities Index

Participation in social and everyday activities were assessed with the Frenchay Activities Index (FAI)^22^. The scale consists of 15 items covering domestic chores, outdoor activities, and leisure/work. Each item is rated from 0 to 3, depending on the frequency of the activity during the past 3 or 6 months. A higher score indicates more frequent participation in social and everyday activities. A total score of < 15 is considered as the person being inactive/restricted^23^.

### Statistical analysis

For descriptive data, mean, standard deviation (SD), median, interquartile range (IQR), minimum, and maximum values were calculated. A cut-off of HADS-D ≥ 4 was used for depression symptoms in bivariate analyses. Group differences between participants with depression symptoms versus no depression symptoms were computed with Chi^2^-test for categorical variables and Mann Whitney *U* for ordinal scale data. Significance level was set to p < 0.05. Eleven hierarchical multiple regression models were performed to investigate the contribution of depression on the dependent variables perceived impact of stroke (each domain of SIS), ADL (BI) and participation in social and everyday activities (FAI), controlling for age, sex, and stroke severity. In the first step, age, sex, and stroke severity were entered simultaneously as independent variables. In the second step, the independent variable depression, as measured by scores on the HADS-D, was added to the model. The models were evaluated by R2, F, F change, and significance level. The Statistical Package for the Social Science software (SPSS version 27, Armonk, NY: IBM Corp) was used.

## Results

**Figure 1:**
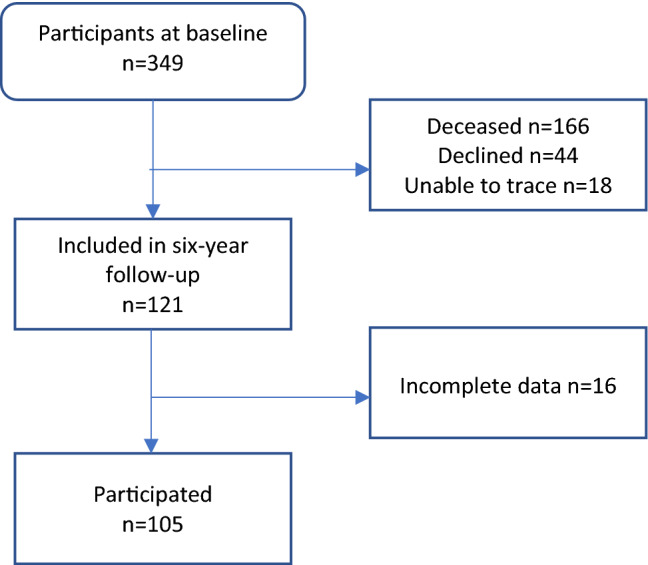
Flow diagram of inclusion of participants.

From the original cohort of 349 individuals in the LAS-1 study, 183 were still alive and eligible for the 6-year follow-up. Of these, 44 individuals declined participation, 18 were not possible to trace, 16 individuals had incomplete data and 105 participated in the current study. The inclusion process is presented in Fig. 1 and demographics of the 105 participants are presented in Table 1. Compared with all 183 individuals that were eligible for the 6-year follow-up, study participants were younger (median age 69 versus 74, p = 0.008). There were no differences between groups with regard to stroke severity (n = 90/10/5 for mild/moderate/severe stroke versus n = 140/25/18, p = 0.055) or sex distribution (57% men versus 54%, p = 0.618).

**Table 1:** Characteristics of participants 6 years after stroke, and comparisons between participants with and without depression symptoms.

Variable	All participants (n = 105)	Persons with HADS < 4 (n = 63)			Persons with HADS ≥ 4 (n = 42)			*p*
	n	n	Median	Min–Max	n	Median	Min–Max	
Age, median (min–max)	69 (30–91)	63	69	30–91	42	72	35–91	0.070
Women/men	45/60	27/36			18/24			1.000
Severity of stroke (at stroke onset) Mild; Moderate; Severe	90/10/5	60/3/0	1	1–2	30/7/5	1	1–3	0.002
Employment (yes/no)	26/79							
Accommodation/Housing Own; Other	101/4							
Civil status Married/Civil partnership; Single	55/50							
Walking ability Walker with/without help/device; Wheelchair user	103/2							
Aphasia (stroke onset) Limited vocabulary; More than yes/no; Yes/no	4/2/2	4/0/0			0/2/2			0.069
Mini Mental State Examination	92	57	29	22–30	35	28	13–30	0.031
Hospital Anxiety and Depression Scale—Depression		63	1	0–3	42	6	4–12	–
Stroke Impact Scale—Strength^a^		62	88	25–100	41	63	13–100	< 0.001
Stroke Impact Scale—Hand function^a^		62	96	43–100	41	79	25–100	< 0.001
Stroke Impact Scale—ADL/IADL^a^		62	89	58–100	41	67	6–97	< 0.001
Stroke Impact Scale—Mobility^a^		62	96	39–100	41	89	25–100	0.027
Stroke Impact Scale—Communication^a^		62	98	40–100	41	78	8–100	< 0.001
Stroke Impact Scale—Emotion^a^		62	97	33–100	41	81	11–100	< 0.001
Stroke Impact Scale—Memory^a^		62	90	0–100	41	85	0–100	0.074
Stroke Impact Scale—Participation^a^		62	92	31–100	41	75	16–100	< 0.001
Stroke Impact Scale—Stroke recovery^a^		63	85	20–100	40	60	0–100	< 0.001
Barthel Index		63	100	75–100	42	95	0–100	< 0.001
Frenchay Activities Index^b^		61	35	8–45	40	21	0–45	< 0.001

^a^n = 103; ^b^n = 101.

The participants were 45 women and 60 men, aged 30–91 (median = 69) at the time of the follow-up. Most of the participants had had a mild stroke (mild n = 90, moderate n = 10, severe n = 5). Among the participants, HADS-D scores varied between 0 and 12 (mean 3.25, SD 3.15, median 2, IQR 1–5). Forty percent had symptoms of depression (HADS ≥ 4) at the 6-year follow-up. The ratings are presented in Table 2.

**Table 2:** Ratings on Hospital Anxiety and Depression Scale-Depression items.

Items:	Persons with HADS-D < 4 (n = 63)		Persons with HADS-D ≥ 4 (n = 42)	
	Mean (SD)	Median (IQR)	Mean (SD)	Median (IQR)
2. Enjoy things	0.2 (0.5)	0 (0–0)	1.0 (0.9)	1 (0–1)
4. Laugh /Funny side	0.1 (0.3)	0 (0–0)	1.0 (0.8)	1 (0–1)
6. Cheerful	0.1 (0.2)	0 (0–0)	0.8 (0.8)	1 (0–1)
8. Slowed down	0.3 (0.4)	0 (0–1)	0.6 (0.8)	0 (0–1)
10. Appearance	0.4 (0.6)	0 (0–1)	1.0 (0.9)	1 (0–2)
12. Look forward with enjoyment	0.1 (0.4)	0 (0–0)	0.5(0.8)	0 (0–1)
14. Book/Radio/TV	0.1 (0.3)	0 (0–0)	0.4 (0.6)	0 (0–1)

In bivariate analyses, there were no differences regarding age, sex, or presence of aphasia between participants with or without depression symptoms (Table 1). However, participants with depression symptoms had greater cognitive impairment, as reflected by lower scores on the MMSE. Participants with depression symptoms also perceived higher impact of stroke on all domains of the SIS, except for the Memory and Thinking, and reported greater difficulties in ADL and social and everyday participation, as reflected by lower scores on BI and FAI (Table 1).

The hierarchical multiple regression models are presented in Table 3 (Supplementary Table S1 online for a more detailed description). Model 1, i.e., age, sex, and stroke severity, was significantly associated with all dependent variables, except the SIS domain ADL. Model 1 explained 4–35% of the variance of the dependent variables. When depression was added (Model 2), the explained variance increased significantly in all models, with ΔR2 ranging from 8 to 54% in increased explained variance of the dependent variables. All models were statistically significant in the second step. Hence depression symptoms were associated with higher perceived impact of stroke in all the domains of SIS, as well as more dependence in ADL as per BI and restrictions in participation measured by FAI.

**Table 3:** The contribution of depression symptoms on perceived impact of stroke (each domain of Stroke Impact Scale; SIS), ADL (Barthel Index) and participation in social and everyday activities (Frenchay Activities Index) (n = 103).

Dependent variable	Model 1 Independent variables: age, sex, stroke severity			Model 2 Independent variables: age, sex, stroke severity, and depression		
	*R*^2^	*F*	*p*	*R*^2^	*ΔF*	*p*
SIS—Strength	0.157	6.160	0.001	0.307	21.153	< 0.001
SIS—Hand function	0.104	3.846	0.012	0.285	24.733	< 0.001
SIS—ADL	0.040	1.364	0.258	0.575	123.438	< 0.001
SIS—Mobility	0.104	3.812	0.012	0.186	9.952	0.002
SIS—Communication	0.262	11.728	< 0.001	0.393	21.195	< 0.001
SIS—Emotion	0.160	6.303	0.001	0.241	10.396	0.002
SIS—Memory and Thinking	0.088	3.196	0.027	0.190	12.319	0.001
SIS—Participation	0.191	7.809	< 0.001	0.357	25.326	< 0.001
SIS—Recovery	0.084	3.041	0.032	0.320	33.870	< 0.001
Barthel Index^a^	0.141	5.510	0.002	0.243	13.499	< 0.001
Frenchay Activities Index^b^	0.351	17.487	< 0.001	0.474	22.503	< 0.001

^a^n = 105. ^b^n = 101.

## Discussion

This study showed that depression symptoms (HADS-D ≥ 4) were present in 40% of the participants in this 6-year follow-up after stroke. Most participants had suffered from a mild stroke, and most had only mild symptoms of depression. Still, depression symptoms were consistently associated with worse outcomes in perceived impact of stroke, as well as more restrictions in ADL, and social and everyday participation. The study adds to the current literature on long-term follow-up^6,12^ showing that depression symptoms are common also in the chronic phase after stroke.

The depressive symptoms were negatively associated with perceived impact of stroke, ADL, and participation in social and everyday activities. Therefore, the clinical implications of depression symptoms after stroke seems important to evaluate. An aspect to take into account is the similarities between depression and apathy. A recent study argued that post-stroke apathy is often mistaken for depression in PSD studies^24^. However, in this study most depression items, that were prominent among the participants with depression symptoms, were clearly mood items (HADS-D item 2, 4, and 6, see Table 2) and not symptoms that could as well be expressions of e.g., lack of energy or post-stroke apathy. Furthermore, participants with depression symptoms had lower scores on the MMSE and the relationship between cognitive function and depression symptoms after stroke could be a relevant focus for future research.

The HADS-D scores in the current study were similar to those reported in a representative sample of the Swedish population in the age group 65–80^25^, indicating that depression symptoms are equally common in the general population. However, whether the negative associations between depressive symptoms, and ADL and participation in social and everyday activities are present also in the general population is not known and should be explored.

The finding of the negative impact of mild depression symptoms on several outcome measures raises the question of how such symptoms might be prevented or treated. In a previous intervention review, evidence for antidepressant or psychological interventions in preventing depression after stroke was weak^11^. However, a systematic review of cognitive behavioural therapy (CBT) for PSD showed positive effects of CBT, but the results should be interpreted with caution due to the quality of the included studies^26^. Another systematic review and meta-analysis found evidence that exercise reduced depressive symptoms in neurologic disorders^27^. Moreover, behavioural activation for PSD has been tested in a few studies with promising results, but more research is needed and recommended especially for milder forms of depression^28^. Thus, for mild depression symptoms after stroke, interventions to test in future studies might include exercise or behavioural interventions, which have few side effects and are easily applied at low cost.

There were several methodological strengths of the study. The follow-up time of 6 years exceeded most previous studies of PSD. All stroke patients from Karolinska University Hospital’s stroke units were eligible for inclusion in the original study and of these, all who were alive and reachable were invited to the 6-year follow-up. Data collection was performed with face-to-face interviews and no exclusion criteria were used, which enabled a broad participation. Valid and reliable outcome measures were used and included patient-reported outcomes. There were also several limitations of the study. First, the sample size was quite small and might not be representative for all persons 6 year after stroke. However, the percentage of deceased participants at the 6-year follow-up was similar to a large Swedish register-based study^29^, which indicates that the original sample was approximately representative for the general stroke population in Sweden 6 years after stroke. Those who chose to participate in the 6-year follow-up were younger than the whole group of eligible individuals alive after 6 years, which limits the representativity of the sample. Second, causal relationships cannot be established i.e., other life events than stroke may have contributed to the depression symptoms identified in this study. Furthermore, it is not possible to establish a causal relationship between the investigated variables, e.g., restrictions in participation might lead to depression and vice versa. However, as depression symptoms were associated with worse functioning, they are important to target.

## Conclusion

The study adds to the literature by providing a long-term follow-up of depression symptoms after stroke and indicating a negative impact of even mild depression symptoms on everyday activities. Hence, long-term follow-up of persons with stroke, with sensitive screening to identify depression symptoms to initiate treatment is warranted.

## Supplementary Information

Supplementary Table S1.

## Data Availability

Since data can indirectly be traced back to the study participants, according to the Swedish and EU personal data sharing legislation, access can only be granted upon request. Request for access to the data can be put to our Research Data Office (rdo@ki.se) at Karolinska Institutet and will be handled according to the relevant legislation.

## Abbreviations

ADL: Activities of daily living
BI: Barthel Index
CBT: Cognitive behavioural therapy
FAI: Frenchay Activities Index
HADS: Hospital Anxiety and Depression Scale
HADS-A: Hospital Anxiety and Depression Scale—Anxiety subscale
HADS-D: Hospital Anxiety and Depression Scale—Depression subscale
ICF: International Classification of Functioning, Disability and Health
IQR: Interquartile range
LAS-1: Life After Stroke Phase 1
MMSE: Mini Mental State Examination
PSD: Post-stroke depression
SD: Standard deviation
SIS: Stroke Impact Scale
