# Supplementary material for: Depression symptoms 6 years after stroke are associated with higher perceived impact of stroke, limitations in ADL and restricted participation
Source: Sci Rep. 2022 May 12;12:7816. doi: 10.1038/s41598-022-11097-9 (PMC9098872; doi:10.1038/s41598-022-11097-9)
Supplement: Supplementary file 1 — Supplementary Table S1. [file 41598_2022_11097_MOESM1_ESM.docx]

Supplementary Information to manuscript:

**Depression symptoms six years after stroke are associated with higher perceived impact of stroke, limitations in ADL and restricted participation**

Charlotte Ytterberg^1,2^, Linda Cegrell^3^, Lena von Koch^1,2^, Maria Wiklander^1^

1. Department of Neurobiology, Care Sciences and Society, Karolinska Institutet, Huddinge, Sweden
2. Karolinska University Hospital, Stockholm, Sweden
3. Department of Physiotherapy, Capio S:t Görans Hospital, Stockholm, Sweden

Corresponding author:

Maria Wiklander

Department of Neurobiology, Care Sciences and Society,
Division of Nursing,
Karolinska Institutet
23 100
SE-141 83 Huddinge
Sweden

Phone. +46852483901

maria.wiklander@ki.se

Supplementary Table S1. Hierarchical multiple regression analyses of contribution of depression symptoms on each domain of Stroke Impact Scale; SIS, Barthel Index; BI, and Frenchay Activities Index; FAI respectively, controlling for age, sex, and stroke severity, six years after onset of stroke (n=103).

| ***Variables*** | ***Model 1*** | ***Unstandardized coefficients*** | | ***Standardized coefficients*** | | |  |  |  | ***Model 2*** | ***Unstandardized coefficients*** | | ***Standardized coefficients*** | | |  |  |  |
| --- | --- | --- | --- | --- | --- | --- | --- | --- | --- | --- | --- | --- | --- | --- | --- | --- | --- | --- |
|  |  | ***B*** | ***SE*** | ***β*** | ***t*** | ***p*** | ***R^2^*** | ***F*** | ***p*** |  | ***B*** | ***SE*** | ***β*** | ***t*** | ***p*** | ***R^2^*** | ***ΔF*** | ***p*** |
| **SIS- Strength** |  |  |  |  |  |  | 0.157 | 6.160 | 0.001 |  |  |  |  |  |  | 0.307 | 21.153 | <0.001 |
| Constant |  | 107.205 | 13.067 |  | 8.204 | <0.001 |  |  |  |  | 103.409 | 11.939 |  | 8.661 | <0.001 |  |  |  |
| Age |  | -0.242 | 0.164 | -0.137 | -1.478 | 0.143 |  |  |  |  | -0.149 | 0.151 | -0.084 | -0.984 | 0.327 |  |  |  |
| Sex |  | 5.362 | 4.539 | 0.110 | 1.182 | 0.240 |  |  |  |  | 6.024 | 4.140 | 0.124 | 1.455 | 0.149 |  |  |  |
| Stroke severity |  | -16.369 | 4.499 | -0.338 | -3.638 | <0.001 |  |  |  |  | -10.176 | 4.317 | -0.210 | -2.357 | 0.020 |  |  |  |
| HADS-D |  |  |  |  |  |  |  |  |  |  | -3.139 | 0.682 | -0.411 | -4.599 | <0.001 |  |  |  |
|  |  |  |  |  |  |  |  |  |  |  |  |  |  |  |  |  |  |  |
| **SIS- Hand function** |  |  |  |  |  |  | 0.104 | 3.846 | 0.012 |  |  |  |  |  |  | 0.285 | 24.733 | <0.001 |
| Constant |  | 107.450 | 8.831 |  | 12.167 | <0.001 |  |  |  |  | 104.717 | 7.951 |  | 13.171 | <0.001 |  |  |  |
| Age |  | -0.111 | 0.111 | -0.095 | -0.997 | 0.321 |  |  |  |  | -0.043 | 0.100 | -0.037 | -0.428 | 0.670 |  |  |  |
| Sex |  | -4.458 | 3.067 | -0.140 | -1.453 | 0.149 |  |  |  |  | -3.982 | 2.757 | -0.125 | -1.444 | 0.152 |  |  |  |
| Stroke severity |  | -9.352 | 3.041 | -0.294 | -3.075 | 0.003 |  |  |  |  | -4.893 | 2.875 | -0.154 | -1.702 | 0.092 |  |  |  |
| HADS-D |  |  |  |  |  |  |  |  |  |  | -2.260 | 0.454 | -0.451 | -4.973 | <0.001 |  |  |  |
|  |  |  |  |  |  |  |  |  |  |  |  |  |  |  |  |  |  |  |
| **SIS-ADL** |  |  |  |  |  |  | 0.040 | 1.364 | 0.258 |  |  |  |  |  |  | 0.575 | 123.438 | <0.001 |
| Constant |  | 92.911 | 10.443 |  | 8.897 | <0.001 |  |  |  |  | 87.536 | 6.999 |  | 12.506 | <0.001 |  |  |  |
| Age |  | -0.148 | 0.131 | -0.112 | -1.127 | 0.262 |  |  |  |  | -0.015 | 0.088 | -0.011 | -0.168 | 0.867 |  |  |  |
| Sex |  | 3.266 | 3.627 | 0.090 | 0.900 | 0.370 |  |  |  |  | 4.204 | 2.427 | 0.115 | 1.732 | 0.086 |  |  |  |
| Stroke severity |  | -4.355 | 3.596 | -0.120 | -1.211 | 0.229 |  |  |  |  | 4.415 | 2.531 | 0.122 | 1.744 | 0.084 |  |  |  |
| HADS-D |  |  |  |  |  |  |  |  |  |  | -4.445 | 0.400 | -0.777 | -11.110 | <0.001 |  |  |  |
|  |  |  |  |  |  |  |  |  |  |  |  |  |  |  |  |  |  |  |
| **SIS- Mobility** |  |  |  |  |  |  | 0.104 | 3.812 | 0.012 |  |  |  |  |  |  | 0.186 | 9.952 | 0.002 |
| Constant |  | 106.218 | 9.520 |  | 11.157 | <0.001 |  |  |  |  | 104.225 | 9.139 |  | 11.405 | <0.001 |  |  |  |
| Age |  | -0.099 | 0.119 | -0.079 | -0.831 | 0.408 |  |  |  |  | -0.050 | 0.115 | -0.040 | -0.433 | 0.666 |  |  |  |
| Sex |  | 1.039 | 3.307 | 0.030 | 0.314 | 0.754 |  |  |  |  | 1.386 | 3.169 | 0.040 | 0.438 | 0.663 |  |  |  |
| Stroke severity |  | -10.456 | 3.278 | -0.305 | -3.190 | 0.002 |  |  |  |  | -7.205 | 3.304 | -0.210 | -2.181 | 0.032 |  |  |  |
| HADS-D |  |  |  |  |  |  |  |  |  |  | -1.648 | 0.522 | -0.305 | -3.155 | 0.002 |  |  |  |
|  |  |  |  |  |  |  |  |  |  |  |  |  |  |  |  |  |  |  |
| **SIS- Communication** |  |  |  |  |  |  | 0.262 | 11.728 | <0.001 |  |  |  |  |  |  | 0.393 | 21.195 | <0.001 |
| Constant |  | 123.750 | 10.716 |  | 11.548 | <0.001 |  |  |  |  | 120.635 | 9.790 |  | 12.323 | <0.001 |  |  |  |
| Age |  | -0.313 | 0.134 | -0.202 | -2.329 | 0.022 |  |  |  |  | -0.236 | 0.124 | -0.153 | -1.910 | 0.059 |  |  |  |
| Sex |  | 5.259 | 3.722 | 0.123 | 1.413 | 0.161 |  |  |  |  | 5.803 | 3.394 | 0.136 | 1.710 | 0.091 |  |  |  |
| Stroke severity |  | -18.380 | 3.690 | -0.432 | -4.981 | <0.001 |  |  |  |  | -13.298 | 3.539 | -0.313 | -3.757 | <0.001 |  |  |  |
| HADS-D |  |  |  |  |  |  |  |  |  |  | -2.576 | 0.560 | -0.384 | -4.604 | <0.001 |  |  |  |
|  |  |  |  |  |  |  |  |  |  |  |  |  |  |  |  |  |  |  |
| **SIS- Emotion** |  |  |  |  |  |  | 0.160 | 6.303 | 0.001 |  |  |  |  |  |  | 0.241 | 10.396 | 0.002 |
| Constant |  | 116.453 | 10.972 |  | 10.614 | <0.001 |  |  |  |  | 114.110 | 10.510 |  | 10.857 | <0.001 |  |  |  |
| Age |  | -0.356 | 0.138 | -0.239 | -2.586 | 0.011 |  |  |  |  | -0.298 | 0.133 | -0.201 | -2.245 | 0.027 |  |  |  |
| Sex |  | 5.843 | 3.811 | 0.143 | 1.533 | 0.128 |  |  |  |  | 6.251 | 3.644 | 0.153 | 1.715 | 0.089 |  |  |  |
| Stroke severity |  | -10.468 | 3.778 | -0.257 | -2.771 | 0.007 |  |  |  |  | -6.647 | 3.800 | -0.163 | -1.749 | 0.083 |  |  |  |
| HADS-D |  |  |  |  |  |  |  |  |  |  | -1.937 | 0.601 | -0.301 | -3.224 | 0.002 |  |  |  |
|  |  |  |  |  |  |  |  |  |  |  |  |  |  |  |  |  |  |  |
| **SIS- Memory and Thinking** |  |  |  |  |  |  | 0.088 | 3.196 | 0.027 |  |  |  |  |  |  | 0.190 | 12.319 | 0.001 |
| Constant |  | 103.841 | 17.601 |  | 5.900 | <0.001 |  |  |  |  | 99.786 | 16.714 |  | 5.970 | <0.001 |  |  |  |
| Age |  | -0.143 | 0.221 | -0.062 | -0.646 | 0.520 |  |  |  |  | -0.043 | 0.211 | -0.019 | -0.202 | 0.841 |  |  |  |
| Sex |  | 4.071 | 6.114 | 0.065 | 0.666 | 0.507 |  |  |  |  | 4.778 | 5.795 | 0.076 | 0.825 | 0.412 |  |  |  |
| Stroke severity |  | -17.213 | 6.061 | -0.274 | -2.840 | 0.005 |  |  |  |  | -10.598 | 6.043 | -0.169 | -1.754 | 0.083 |  |  |  |
| HADS-D |  |  |  |  |  |  |  |  |  |  | -3.353 | 0.955 | -0.339 | -3.510 | 0.001 |  |  |  |
|  |  |  |  |  |  |  |  |  |  |  |  |  |  |  |  |  |  |  |
| **SIS- Participation** |  |  |  |  |  |  | 0.191 | 7.809 | <0.001 |  |  |  |  |  |  | 0.357 | 25.326 | <0.001 |
| Constant |  | 100.809 | 10.960 |  | 9.198 | <0.001 |  |  |  |  | 97.385 | 9.844 |  | 9.893 | <0.001 |  |  |  |
| Age |  | -0.112 | 0.138 | -0.074 | -0.811 | 0.419 |  |  |  |  | -0.027 | 0.124 | -0.018 | -0.217 | 0.829 |  |  |  |
| Sex |  | 6.401 | 3.807 | 0.154 | 1.681 | 0.096 |  |  |  |  | 6.998 | 3.413 | 0.168 | 2.050 | 0.043 |  |  |  |
| Stroke severity |  | -15.898 | 3.774 | -0.383 | -4.212 | <0.001 |  |  |  |  | -10.312 | 3.559 | -0.248 | -2.897 | 0.005 |  |  |  |
| HADS-D |  |  |  |  |  |  |  |  |  |  | -2.832 | 0.563 | -0.433 | -5.032 | <0.001 |  |  |  |
|  |  |  |  |  |  |  |  |  |  |  |  |  |  |  |  |  |  |  |
| **SIS- Recovery** |  |  |  |  |  |  | 0.084 | 3.041 | 0.032 |  |  |  |  |  |  | 0.320 | 33.870 | <0.001 |
| Constant |  | 96.384 | 13.896 |  | 6.936 | <0.001 |  |  |  |  | 92.387 | 12.059 |  | 7.661 | <0.001 |  |  |  |
| Age |  | -0.140 | 0.177 | -0.076 | -0.792 | 0.430 |  |  |  |  | -0.033 | 0.154 | -0.018 | -0.214 | 0.831 |  |  |  |
| Sex |  | 1.805 | 4.913 | 0.036 | 0.367 | 0.714 |  |  |  |  | 2.439 | 4.258 | 0.048 | 0.573 | 0.568 |  |  |  |
| Stroke severity |  | -13.516 | 4.818 | -0.271 | -2.805 | 0.006 |  |  |  |  | -5.496 | 4.396 | -0.110 | -1.250 | 0.214 |  |  |  |
| HADS-D |  |  |  |  |  |  |  |  |  |  | -4.089 | 0.703 | -0.515 | -5.820 | <0.001 |  |  |  |
|  |  |  |  |  |  |  |  |  |  |  |  |  |  |  |  |  |  |  |
| **BI^a^** |  |  |  |  |  |  | 0.141 | 5.510 | 0.002 |  |  |  |  |  |  | 0.243 | 13.499 | <0.001 |
| Constant |  | 105.572 | 7.795 |  | 13.543 | <0.001 |  |  |  |  | 104.037 | 7.365 |  | 14.125 | <0.001 |  |  |  |
| Age |  | -0.055 | 0.099 | -0.052 | -0.561 | 0.576 |  |  |  |  | -0.013 | 0.094 | -0.012 | -0.134 | 0.894 |  |  |  |
| Sex |  | 4.834 | 2.739 | 0.164 | 1.765 | 0.081 |  |  |  |  | 5.050 | 2.584 | 0.171 | 1.954 | 0.053 |  |  |  |
| Stroke severity |  | -9.172 | 2.705 | -0.314 | -3.391 | 0.001 |  |  |  |  | -6.120 | 2.684 | -0.210 | -2.280 | 0.025 |  |  |  |
| HADS-D |  |  |  |  |  |  |  |  |  |  | -1.577 | 0.429 | -0.339 | -3.674 | <0.001 |  |  |  |
|  |  |  |  |  |  |  |  |  |  |  |  |  |  |  |  |  |  |  |
| **FAI^b^** |  |  |  |  |  |  | 0.351 | 17.487 | <0.001 |  |  |  |  |  |  | 0.474 | 22.503 | <0.001 |
| Constant |  | 60.343 | 5.331 |  | 11.318 | <0.001 |  |  |  |  | 58.487 | 4.839 |  | 12.086 | <0.001 |  |  |  |
| Age |  | -0.299 | 0.068 | -0.362 | -4.403 | <0.001 |  |  |  |  | -0.252 | 0.062 | -0.305 | -4.048 | <0.001 |  |  |  |
| Sex |  | 0.123 | 1.891 | 0.005 | 0.065 | 0.948 |  |  |  |  | 0.090 | 1.711 | 0.004 | 0.052 | 0.958 |  |  |  |
| Stroke severity |  | -10.242 | 1.864 | -0.452 | -5.494 | <0.001 |  |  |  |  | -7.640 | 1.773 | -0.337 | -4.308 | <0.001 |  |  |  |
| HADS-D |  |  |  |  |  |  |  |  |  |  | -1.387 | 0.292 | -0.375 | -4.744 | <0.001 |  |  |  |

^a^ n=105, ^b^n=101
